# Exploring the role of epigenetic regulation in cancer prognosis with epigenetic score

**DOI:** 10.3389/fphar.2025.1538205

**Published:** 2025-02-18

**Authors:** Ruiguang Zhang, Shimin Jin, Qi Xu, Rongxiao Dai

**Affiliations:** ^1^ Department of Neurosurgery, Jiading District Central Hospital Affiliated Shanghai University of Medicine and Health Sciences, Shanghai, China; ^2^ Department of Gastroenterology, Second Affiliated Hospital of Dalian Medical University, Dalian, China

**Keywords:** epigenetic regulation, cancer prognosis, pan-cancer, prognosis prediction, nomogram

## Abstract

**Background:**

The mechanisms of epigenetic regulation emerge as a fundamental determinant in the complex landscape of cancer initiation and advancement. However, the specific impact of epigenetic regulation on cancer progression remains unclear. To explore the relationship between epigenetic regulation and cancer progression, we utilized transcriptomic data from The Cancer Genome Atlas (TCGA) datasets to investigate the association.

**Methods:**

We obtained transcriptomic data of epigenetic gene dataset from the TCGA database and calculated an epigenetic score using the Least Absolute Shrinkage and Selection Operator (LASSO) Cox model. Additionally, we created a nomogram that integrates the epigenetic score and clinical features, providing a more comprehensive tool for tumor patients prognosis assessment.

**Results:**

We calculated the epigenetic score based on the expression levels of epigenetic-related genes. The nomogram we developed incorporates the epigenetic score and clinical characteristics. The epigenetic score was positively correlated with the expression of genes related to hallmarkers of cancer, including glycolysis, epithelial-mesenchymal transition (EMT), cell cycle, DNA repair, angiogenesis, and inflammatory response. Furthermore, we performed gene ontology (GO) and kyoto encyclopedia of genes and genomes (KEGG) analysis to explore the signaling pathways and biological processes in high epigenetic score group.

**Conclusion:**

The epigenetic scoring system developed in this investigation represents an innovative approach that demonstrates remarkable potential in forecasting survival trajectories across diverse cancer types. These groundbreaking insights not only illuminate the intricate interactions between epigenetic mechanisms and gene expression regulation in oncological contexts, but also indicate that the derived epigenetic metric could potentially emerge as a significant prognostic biomarker for cancer outcomes.

## Introduction

Epigenetics is one of the important mechanisms for regulating gene expression. Epigenetics regulation is characterized by transmitting genetic information without altering the DNA sequence, including DNA methylation, histone modification, microRNA induced gene expression alteration (Zhang et al., 2020). By affecting the expression levels of genes, epigenetic regulation can modulate cell proliferation and differentiation, and thus participate in the occurrence and development of cancer, providing a new perspective for us to explore the mechanisms of cancer development and progression (Dawson and Kouzarides, 2012).

Cancer is a disease caused by uncontrolled cell proliferation, characterized by features such as abnormal cell growth, invasiveness, and evasion of apoptosis (Hanahan, 2022). Extensive research has demonstrated that carcinogenesis is a multifaceted process extending beyond genetic mutations, with epigenetic alterations serving as critical determinants in the complex mechanisms of cancer development and progression (Sharpless and Chin, 2003). Within malignant cellular environments, aberrant DNA methylation patterns frequently result in the suppression of tumor-protective genes while simultaneously facilitating the activation of oncogenic genetic elements (Salgia and Skarin, 1998). For example, the high methylation level of tumor suppressor *p16INK4a* promotor is invloved in the occurrence of melanoma and lung cancer (Sharpless and Chin, 2003; Salgia and Skarin, 1998). In breast cancer, the deacetylation of histone H3K27 in the *BRCA1* gene leads to a compact chromatin structure, thereby suppressing gene transcription and expression (Romagnolo et al., 2015). Furthermore, the long non-coding RNA *HOTAIR* inhibits the expression of tumor suppressor genes and is closely related to the occurrence of breast cancer and gastric cancer (Kong et al., 2022; Cai et al., 2014).

Epigenetic regulatory mechanisms significantly contribute to the complex architectural and functional development of the tumor microenvironment (Dawson and Kouzarides, 2012). The histone H3K4 methylation of the *ras* gene is associated with its activation in pan-cancer, promoting cell proliferation (Shilatifard, 2012). The IL-1β in the tumor microenvironment can induce the acetylation of histone H3, activating the expression of genes related to tumor progression (Han et al., 2023). The hypoxic state in the tumor microenvironment can upregulate the expression of miR-21, promoting the proliferation and metastasis of cancer cells (Angel et al., 2023). In addition, environmental factors such as diet, chemicals, radiation, and viral infections can also cause epigenetic changes, thereby affecting the occurrence and development of tumors. For example, certain dietary components can directly affect the DNA methylation pattern, thereby increasing the risk of cancer (Sapienza and Issa, 2016). On the other hand, viral infections such as human papillomavirus (HPV) have also been found to promote the occurrence of cancer by altering the epigenetic state of the host cells (Fang et al., 2014; Revathidevi et al., 2021).

In summary, epigenetic regulation has become an important field in the study of tumor biology by affecting gene expression and its interactions. The research reveals the nuanced and multifaceted role of epigenetic regulation in cancer development. The impact of epigenetic mechanisms on oncological processes cannot be attributed to a single gene’s expression, but rather emerges from the complex interplay of multiple epigenetic regulatory pathways. Consequently, it would be overly simplistic to categorize epigenetics as uniformly beneficial or detrimental to tumor progression. A comprehensive understanding of these intricate epigenetic regulatory mechanisms offers promising avenues for advancing early cancer detection and developing targeted therapeutic interventions. Leveraging The Cancer Genome Atlas (TCGA) datasets, we have developed an innovative epigenetic scoring system. This novel assessment approach aims to elucidate the prognostic significance of epigenetic gene expression patterns in cancer patients. Furthermore, it provides a new research direction for elucidating the role of epigenetic regulation in cancer development and progression.

## Material and methods

### Identification of epigenetic-related genes

Epigenetic genes were obtained from the literature reported by yan et al. (Zhang et al., 2022). A total of 990 epigenetic genes were summarized in the study (Supplementary Table S1).

### Patients and datasets

The research data were obtained from the TCGA database, downloaded through the UCSC Xena platform (http://xenabroswer.net/hub), comprising 8,739 pan-cancer transcriptomic profiles. The dataset ID is “EB++AdjustPANCAN_IlluminaHiSeq_RNASeqV2.geneExp.xena”. The analysis included pan-cancer data from 32 types of solid tumors, excluding samples of acute myeloid leukemia. To ensure the reliability and generalizability of the model, We divided the samples into a training cohort and a testing cohort by randomly assigning 70% of the samples to the training cohort and the remaining 30% to the testing cohort.

### Construction of the prognostic epigenetic-related signature

The signature construction followed these steps: (1) Based on the training set, we performed univariate Cox regression analysis to screen for prognostic genes significantly associated with survival (*p* < 0.0001, HR ≤ 0.7, HR ≥ 1.3). (2) We then used the Least Absolute Shrinkage and Selection Operator (LASSO) regression algorithm to perform feature selection, reducing the risk of overfitting. (3) Finally, we constructed the final predictive model using a multivariate Cox proportional hazards regression model.

The epigenetic score was trained using all cancer types collectively. The epigenetic score was calculated as follows: Epigenetic Score = Σ(Expression level of gene i × Corresponding regression coefficient). Specifically, Epigenetic Score = 0.147*ACTB+0.372*AP2A1+0.307*ASXL1+-0.14*BAHD1+0.104*BCDIN3D+0.193*BRD4+0.095*CDYL+0.179*DDX17+-0.097*DDX24+-0.278*DDX5+0.079*DHX35 + 0.128*DHX8+0.101*ENY2+0.117*FKBP1A + -0.269*FTSJ1+-0.33*HDGF + -0.196*KDM4B + -0.264*L3MBTL2+-0.183*MEPCE+0.081*PAK2+0.293*PHC2+-0.141*PHF7+-0.177*SETD3+-0.17*SETDB2+-0.158*SETMAR+0.134*SIRT7+0.065*SP140L+0.299*SUPT7L + -0.114*TADA2B+0.17*UBE2A+0.175*UCHL5+-0.103*USP7+-0.45*YTHDC1. Patients were divided into high epigenetic score and low epigenetic score groups based on their epigenetic score: samples with a standardized epigenetic score (z-score) greater than 0 were defined as the high epigenetic score group, while those with a standardized epigenetic score less than or equal to 0 were defined as the low epigenetic score group.

### Construction of the nomogram

The nomogram was constructed using the nomogram function from the rms R package, setting the linear predictor (Linear Predictor) and survival probabilities (1-year, 3-year, 5-year, and 10-year) as output variables, with the maximum score set to 100 points.

### Evaluation of biological processes

The ssgsea method was used to evaluate the activities of key biological processes.The ssgsea was performed using the GSVA R package. The gene sets for glycolysis, EMT, DNA repair, angiogenesis, and inflammatory response were obtained from www.gsea-msigdb.org, including HALLMARK_ANGIOGENESIS, HALLMARK_DNA_REPAIR, HALLMARK_EPITHELIAL_MESENCHYMAL_TRANSITION, HALLMARK_INFLAMMATORY_RESPONSE, and WP_GLYCOLYSIS_IN_SENESCENCE. The gene set for cell cycle was derived from previous study (Sanchez-Vega et al., 2018).

### Differential gene expression analysis

We used the limma R package to analyze the differentially expressed genes (DEGs) between the high-risk and low-risk groups, applying the following selection criteria: log2 fold change absolute value is greater than or equal to 1 and adjusted false discovery rate (FDR) *p*-value less than 0.05.

### Statistical analysis

All statistical analyses were performed using R software (version 4.2.2). Kaplan-Meier analysis was used to evaluate patient survival, and decision curve analysis (DCA) was used to assess the clinical utility of the model. Time-dependent receiver operating characteristic (ROC) curve analysis was used to compare the predictive performance of the ES and the nomogram model (area under the curve, AUC). All statistical tests were two-sided, and *p*-values less than 0.05 were considered statistically significant. In univariate Cox analysis, HR > 1 is considered risk factors, while HR < 1 is considered protective factors.

## Results

### Identification of epigenetic-related signature in pan-cancer

**FIGURE 1:**
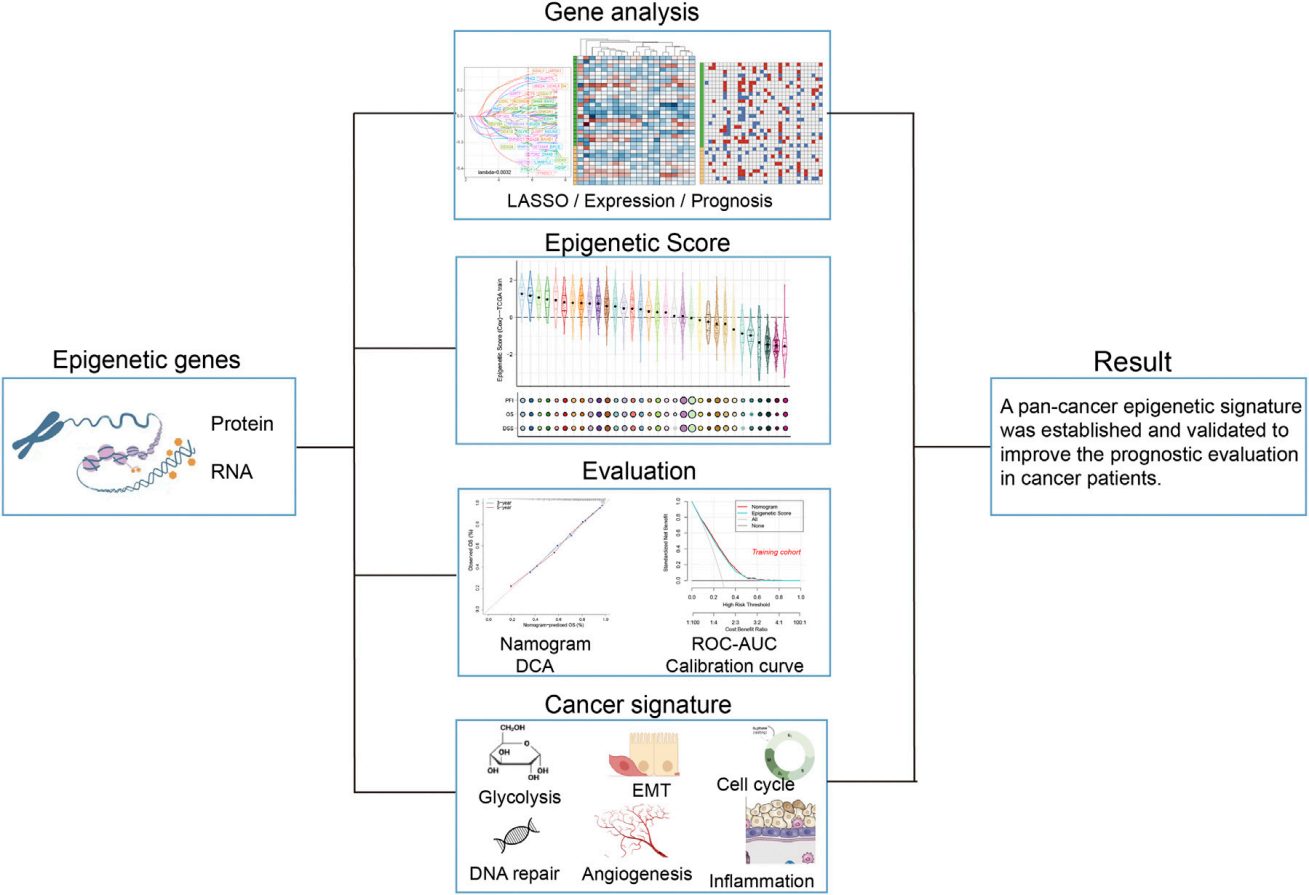
Workflow for analyzing the relationship between epigenetic-related genes and pan-cancer prognosis.

**FIGURE 2:**
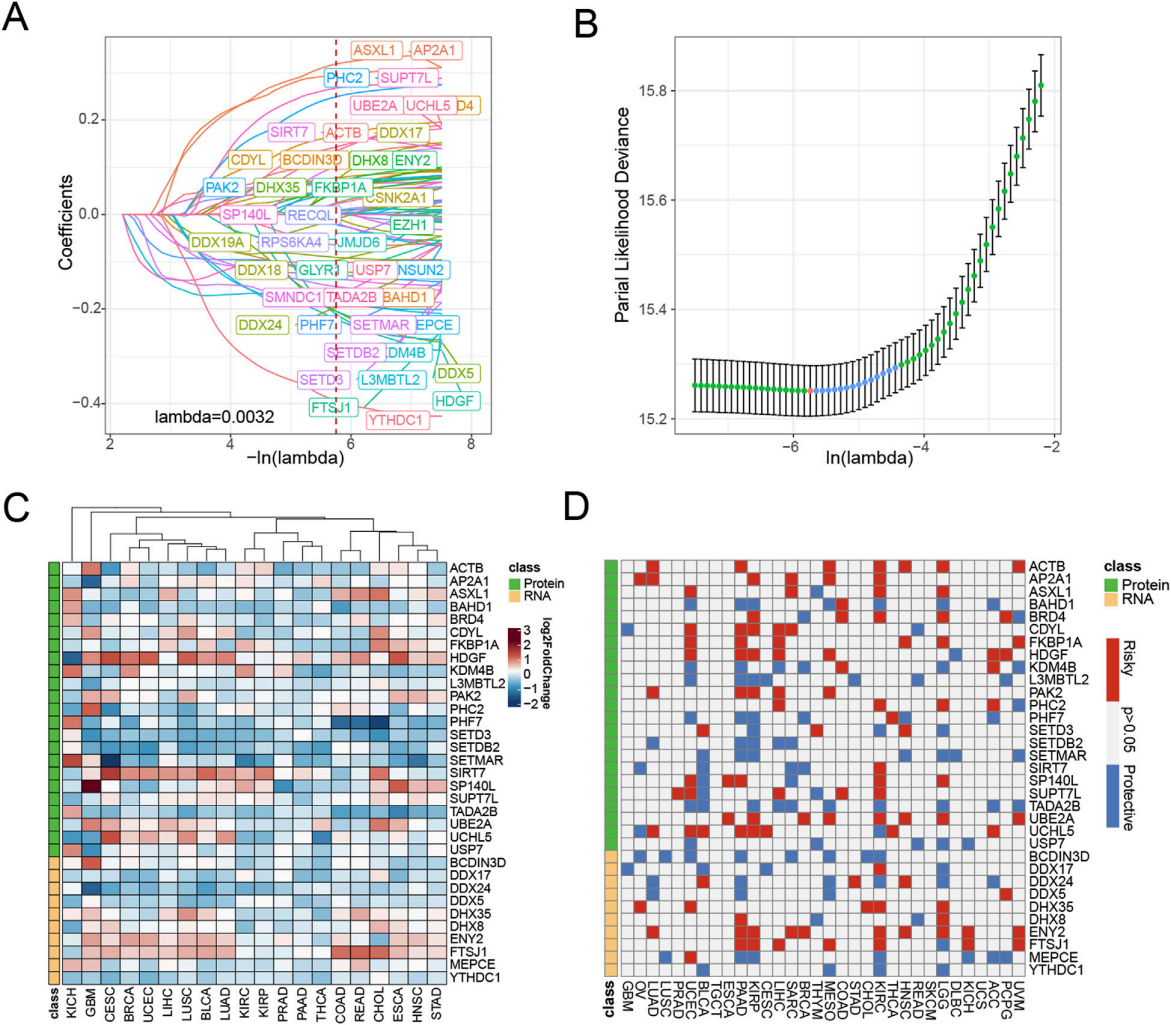
Screening of epigenetic-related genes in pan-cancer. **(A)** LASSO regressions were employed in the epigenetic-related signature identity. Coefficient profile plot of predictors was performed against the log(λ) sequence. **(B)** LASSO regression model cross-validation plot. A vertical line was drawn at the optimum with the minimum criterion. Thirty-three variables were selected when the most available parameter value λ = 0.0032. **(C)** Compared with normal tissues, the expression level of genes in various tumor types according to TCGA datasets. Red indicates genes with high expression in tumors. Blue indicates genes with low expression in tumors. **(D)** Univariable Cox analysis was employed according to TCGA datasets. Red represents genes associated with worse tumor prognosis (HR > 1, indicating higher risk), while blue represents genes associated with better prognosis (HR < 1, indicating a protective effect).

We established a systematic workflow for constructing the epigenetic-related signature of pan-cancer (Figure 1). In the initial screening stage, we performed univariate Cox analysis on 990 epigenetic-related genes and identified 59 candidate genes (*p* < 0.0001, HR ≤ 0.7, HR ≥ 1.3) Supplementary Table S2). To further optimize feature selection, we conducted LASSO regression analysis on these candidate genes using the TCGA pan-cancer training set (Figures 2A, B). Through stepwise Cox proportional hazards regression modeling, we ultimately identified 33 key epigenetic-related genes with important prognostic value, and established an epigenetic score based on the standardized expression levels of these genes.

To elucidate the roles of various epigenetic modifications in cancer progression, we categorize epigenetic genes based on their functions into two groups: protein modification-related genes and RNA modification-related genes. To gain deeper insights into the distinctive characteristics of 33 epigenetic associated biomarkers, we conducted a comprehensive analysis comparing their expression profiles across tumor and corresponding normal tissue specimens. Our investigation aimed to elucidate the molecular variations in these critical biomarkers between cancerous and non-cancerous tissue environments. We discovered that protein modification-related, including *HDGF*, *SIRT-7*, *UBE2A*, and *UCHL5*, elevated expression in tumor tissues. Similarly, the RNA modification-related genes *DHX35*, *DHX8*, *ENY2*, and *FTSJ1* also upregulated in most tumor tissues. These findings suggest that epigenetic gene expression undergoes significant alterations in tumor tissues compared to normal tissues, indicating that epigenetic dysregulation may be a critical mechanism underlying tumor initiation (Figure 2C).

Notably, through univariate Cox analysis (Figure 2D), we found that these epigenetic-related genes did not exhibit clear protective or risk characteristics in most cancer types. This result suggests that epigenetic regulation may play a more complex role in the tumor progression process, rather than a simple promoting or inhibiting relationship. This complexity also highlights the necessity of further investigating the underlying mechanisms of epigenetic regulatory networks in tumor development.

### Landscape of pan-cancer epigenetic scores and their clinical implications

Our systematic analysis of ES across 32 different cancer types revealed significant variations in epigenetic profiles among tumors from different organ origins. Nineteen cancer types, including glioblastoma (GBM), mesothelioma (MESO), esophageal carcinoma (ESCA), cholangiocarcinoma (CHOL), and uterine carcinosarcoma (UCS) and other types exhibited relatively high epigenetic scores. In contrast, 11 malignant neoplasms, such as kidney chromophobe (KICH), prostate adenocarcinoma (PRAD), thyroid carcinoma (THCA), testicular germ cell tumors (TGCT), thymoma (THYM), pheochromocytoma and paraganglioma (PCPG), breast invasive carcinoma (BRCA), uterine corpus endometrial carcinoma (UCEC), kidney renal papillary cell carcinoma (KIRP), uveal melanoma (UVM), and adrenocortical carcinoma (ACC), displayed lower epigenetic scores.

We analyzed the differences in epigenetic scores across different types of tumors (Supplementary Figure S1). Our research findings indicate that in most cancer types, such as bladder cancer (BLCA), breast cancer (BRCA), colon cancer (COAD), gastrointestinal cancer (KIH), Kidney Inferred Carcinoma (KIRC), Kidney Papillary Cell Carcinoma (KIRP), liver cancer (LIHC), lung adenocarcinoma (LUAD), lung squamous cell carcinoma (LUSC), TGCT, and thyroid cancer (THCA), there is a significant upward trend in epigenetic scores as tumor staging increases. This finding suggests a significant correlation between tumor progression and epigenetic characteristics. However, in some tumor types, the epigenetic scores did not show a significant increase with disease progression, which may be related to insufficient clinical samples or missing information. Furthermore, in tumor types such as cervical cancer (CESC) and endometrial cancer (UCEC), the epigenetic scores also exhibit a gradual increase with higher tumor grades. This further supports the close relationship between tumor progression and epigenetic mechanisms.

**FIGURE 3:**
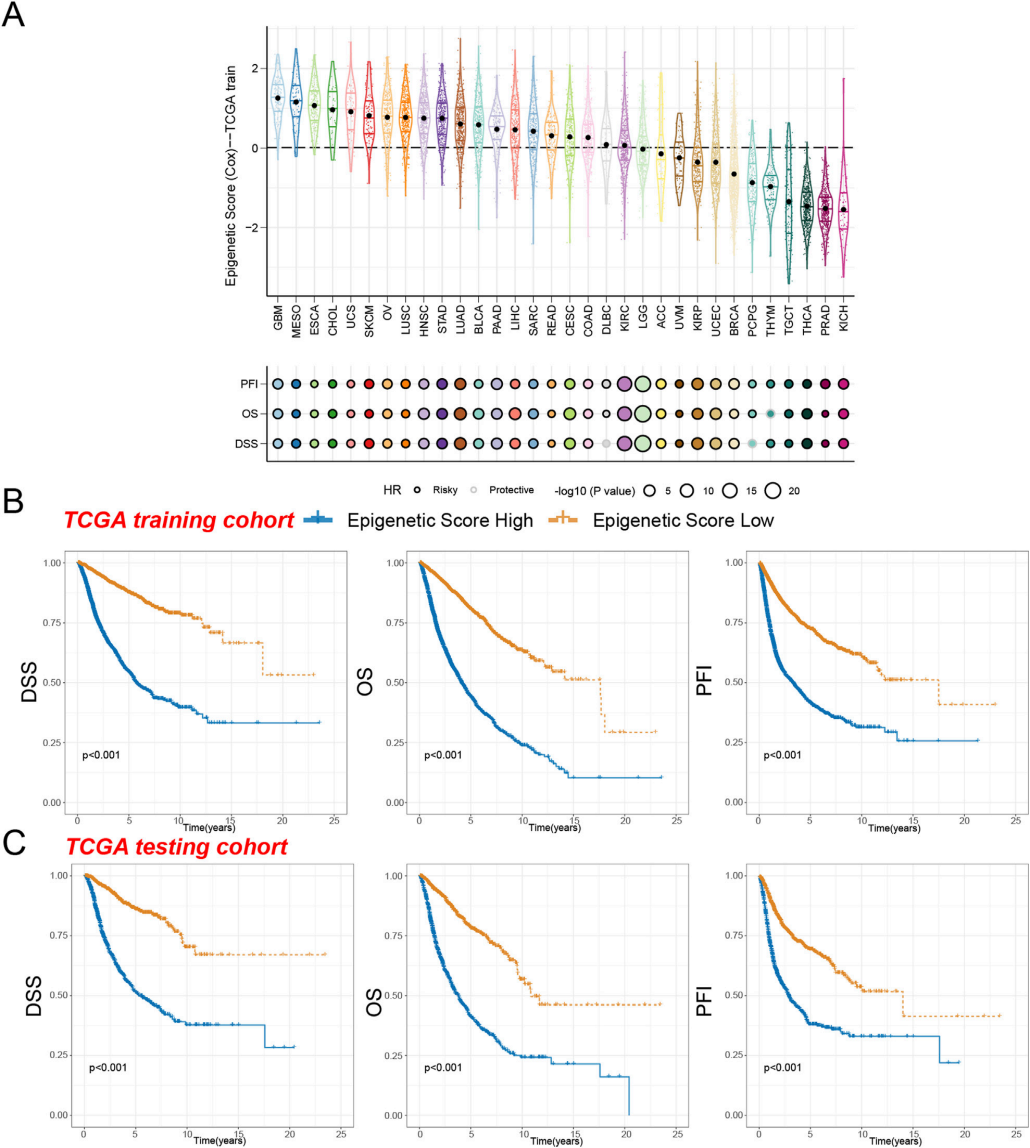
Survival indicators of the epigenetic score in pan-cancer. **(A)** The epigenetic scores of various tumor types in the training cohort. Hazard ratio (HR) of progression-free interval (PFI), overall survival (OS), and disease-specific survival (DSS) in various tumor types were calculated. Circles with a black line indicate a risky effect, while a light gray line indicates a protective effect. The value of hazard ratio can be obtained by circle size. **(B)** Patients in the TCGA training cohort were assigned to high or low epigenetic score groups according to the median epigenetic score. The DSS, OS, and PFI of different patient groups in the training cohort were shown. **(C)** The DSS, OS, and PFI of high and low epigenetic score groups in the test cohort were shown.

Further analysis revealed that the epigenetic score held significant prognostic value across most cancer types (Supplementary Figure S2). In the majority of tumor types, the high epigenetic score acted as a risk factor and was significantly associated with patients’ progression-free interval (PFI), overall survival (OS), and disease-specific survival (DSS). Notably, the high epigenetic score was identified as a risk factor for PFI across all cancer types, with only THYM showing a protective effect in OS analysis and diffuse large B-cell lymphoma (DLBC) and PCPG exhibiting protective roles in DSS analysis. Interestingly, even though KIRC and brain lower grade glioma (LGG) had moderate epigenetic scores, the epigenetic score remained a strong risk factor for predicting the prognosis of these two cancer types (Figure 3A).

To assess the prognostic reliability of our epigenetic score, we segmented patients in the TCGA training cohort into high epigenetic score group and low epigenetic score group. Our analysis revealed that individuals in the high epigenetic score group demonstrated significantly reduced Disease-Specific Survival (DSS), Overall Survival (OS), and Progression-Free Interval (PFI) compared to the low-risk cohort (Figure 3B). These findings were subsequently validated in the independent testing cohort, which consistently demonstrated that the high-risk group experienced markedly inferior survival trajectories (Figure 3C). These results not only confirm the reliable prognostic prediction capability of our epigenetic score but also suggest its potential broad applicability across pan-cancer.

### Establishment and evaluation of a nomogram based on epigenetic scores for predicting patient survival rates

**FIGURE 4:**
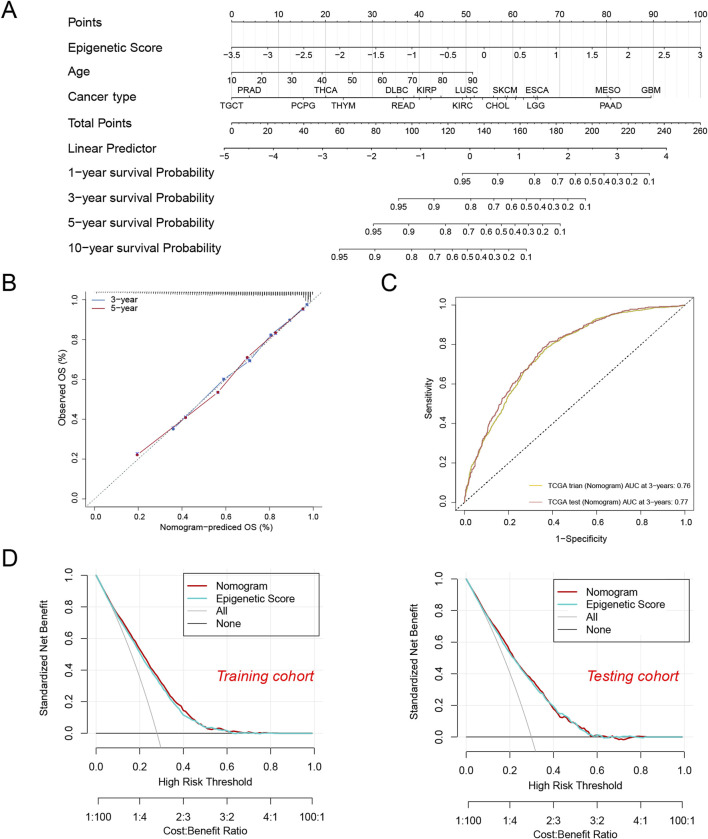
A nomogram were established and evaluated based on the epigenetic scores for predicting the patient’s survival. **(A)** Nomogram to predict the tumor patient survival. Patients’ clinical characteristics and epigenetic score were enrolled in the nomogram. Draw a line perpendicular from the corresponding axis of each risk factor until it reaches the line labeled “Total Points”. Sum up the number of points for all risk factors, then draw a line descending from the axis labeled “survival probability” until it intercepts prognosis probabilities. **(B)** Calibration curves for 3-year and 5-year overall survival (OS) in the training cohort. **(C)** The area under the curve (AUC) values for the nomogram in the training and test cohorts at 3 years. **(D)** Decision curve analysis of the nomogram in the training and testing cohorts.

To translate the epigenetic score into a practical clinical tool, we developed an integrative prediction model. This model was presented in the form of a nomogram (Figure 4A), which not only incorporated the epigenetic score but also integrated key clinical features such as age and tumor type, aiming to provide clinicians with an intuitive and comprehensive prognostic assessment tool.

To validate the reliability of the model, we conducted multi-dimensional performance evaluations. The calibration curve analysis showed that the model’s predictions of 3-year and 5-year overall survival were highly consistent with the actual observations (Figure 4B), confirming the accuracy of the predictions. In terms of predictive efficacy, the model achieved AUC values of 0.76 and 0.77 in the training and testing sets, respectively (Figure 4C), indicating its stable and reliable predictive capability. Further, decision curve analysis (DCA) demonstrated that the integrated model generated positive net benefits across various decision thresholds (Figure 4D). Although the integrated model did not show statistically significant advantages over using the epigenetic score alone, its provision of a multi-dimensional risk assessment is of great clinical relevance. For instance, we found that patients with glioblastoma (GBM) and pancreatic adenocarcinoma (PAAD) had significantly lower expected survival rates compared to those with testicular germ cell tumors (TCGT) and prostate adenocarcinoma (PRAD). This risk stratification based on multiple factors not only helps clinicians identify high-risk patients but also provides a scientific basis for developing personalized treatment strategies.

In summary, the integration model of epigenetic scores and clinical features can significantly improve the accuracy of prognostic prediction and provide clinicians with a reliable decision-support tool. These findings emphasize the important value ofepigenetic score in modern precision oncology.

### Epigenetic signature and biological characteristic in pan-cancer

The development of malignant tumors is a complex biological transformation process. During tumor progression, normal cells may acquire a series of key characteristics, including increased glycolysis, EMT, sustained cell proliferation and so on. To deeply explore the intrinsic connection between epigenetic regulation and tumor biological features, we employed the ssgsea algorithm to systematically quantify the activities of cancer-related pathways in pan-cancer.

**FIGURE 5:**
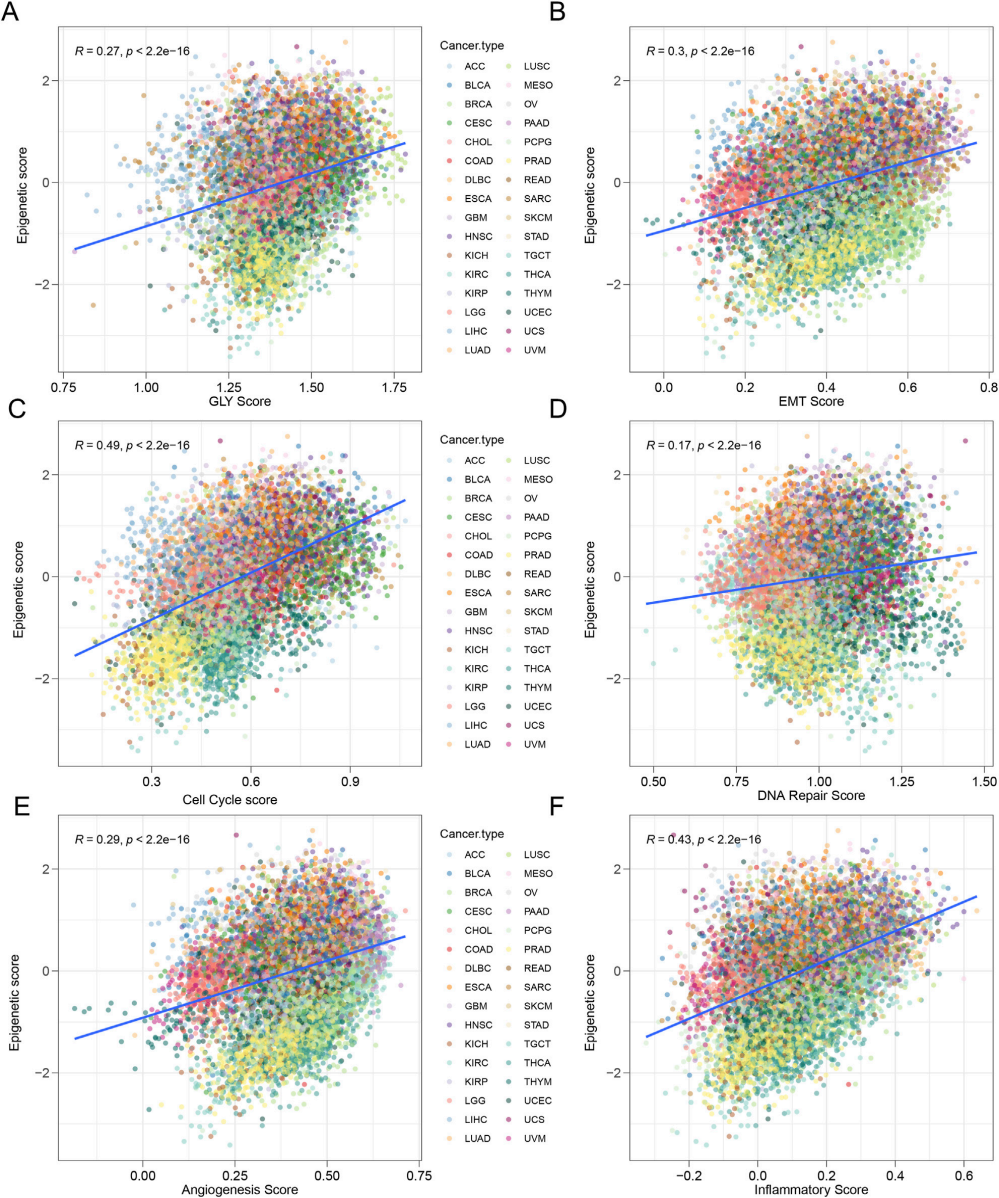
Tumors with high epigenetic scores represent an aggressive phenotype. **(A)** The activity of glycolysis-related pathways was positively correlated with epigenetic score in various tumor types. *R* = 0.27, *p* < 0.0001. **(B)** The activity of epithelial-to-mesenchymal transition (EMT) related pathways was positively correlated with senescence score. *R* = 0.3, *p* < 0.001. **(C)** The activity of cell cycle-related pathways was positively correlated with epigenetic score. *R* = 0.49, *p* < 0.0001. **(D)** The activity of DNA repair related pathways was positively correlated with epigenetic score. *R* = 0.17, *p* < 0.0001. **(E)** The activity of anginogenesis related pathways was positively correlated with epigenetic score. *R* = 0.29, *p* < 0.0001. **(F)** The activity of inflammatory related pathways was positively correlated with epigenetic score. *R* = 0.43, *p* < 0.0001.

Through comprehensive analysis of pan-cancer data, we found that the epigenetic score exhibited significant positive correlations with key tumor biological processes. Specifically, the epigenetic score was positively correlated with the expression of genes related to glycolysis (*R* = 0.27, *p* < 0.001), EMT (*R* = 0.3, *p* < 0.001), and cell cycle (*R* = 0.49, *p* < 0.001), DNA repair (*R* = 0.17, *p* < 0.001), angiogenesis (*R* = 0.29, *p* < 0.001), inflammation (*R* = 0.43, *p* < 0.001) (Figure 5). This funding reveals that tumors with elevated epigenetic scores are associated with enhanced metabolic, cellular, and microenvironmental characteristics, including increased glycolytic metabolism, more aggressive EMT, higher cell cycle proliferation, improved DNA repair mechanisms, enhanced angiogenesis, and heightened inflammatory response. These features indicate an aggressive phenotype.

In further cancer type specific analyses, we observed interesting differences. In most tumor types, the expression of EMT-related genes was positively correlated with the epigenetic score, but in a few tumor types, such as cholangiocarcinoma (CHOL), melanoma (SKCM), and uterine carcinosarcoma (UCS), this relationship was negatively correlated (Supplementary Figure S3). The expression patterns of genes related to glycolysis, cell cycle, DNA repair, angiogenesis, inflammatory response also showed complex variability, with most tumor types exhibiting positive correlations between gene expression and epigenetic score (Supplementary Figures S3–5). It is worth noting that the correlation between epigenetic scores and tumor biological characteristics varies across different types of cancer. Different tumor types exhibit unique epigenetic regulation patterns, and this heterogeneity reflects the complexity and individual differences of epigenetic regulation in pan-cancer.

### Functional analysis of tumor samples stratified by epigenetic scores

Our comprehensive analysis revealed that tumors characterized by elevated epigenetic scores demonstrated more aggressive and invasive biological properties. A critical research objective was to determine whether the biological mechanisms underlying the high-risk group were intrinsically linked to tumor invasion processes. To elucidate these potential correlations, we systematically divided the samples into two distinct groups based on their epigenetic score stratification and conducted an extensive functional enrichment analysis. This investigation encompassed a multifaceted approach, including KEGG pathway annotations, biological process (BP) characterizations, cellular component (CC) assessments, and molecular function (MF) evaluations.

**FIGURE 6:**
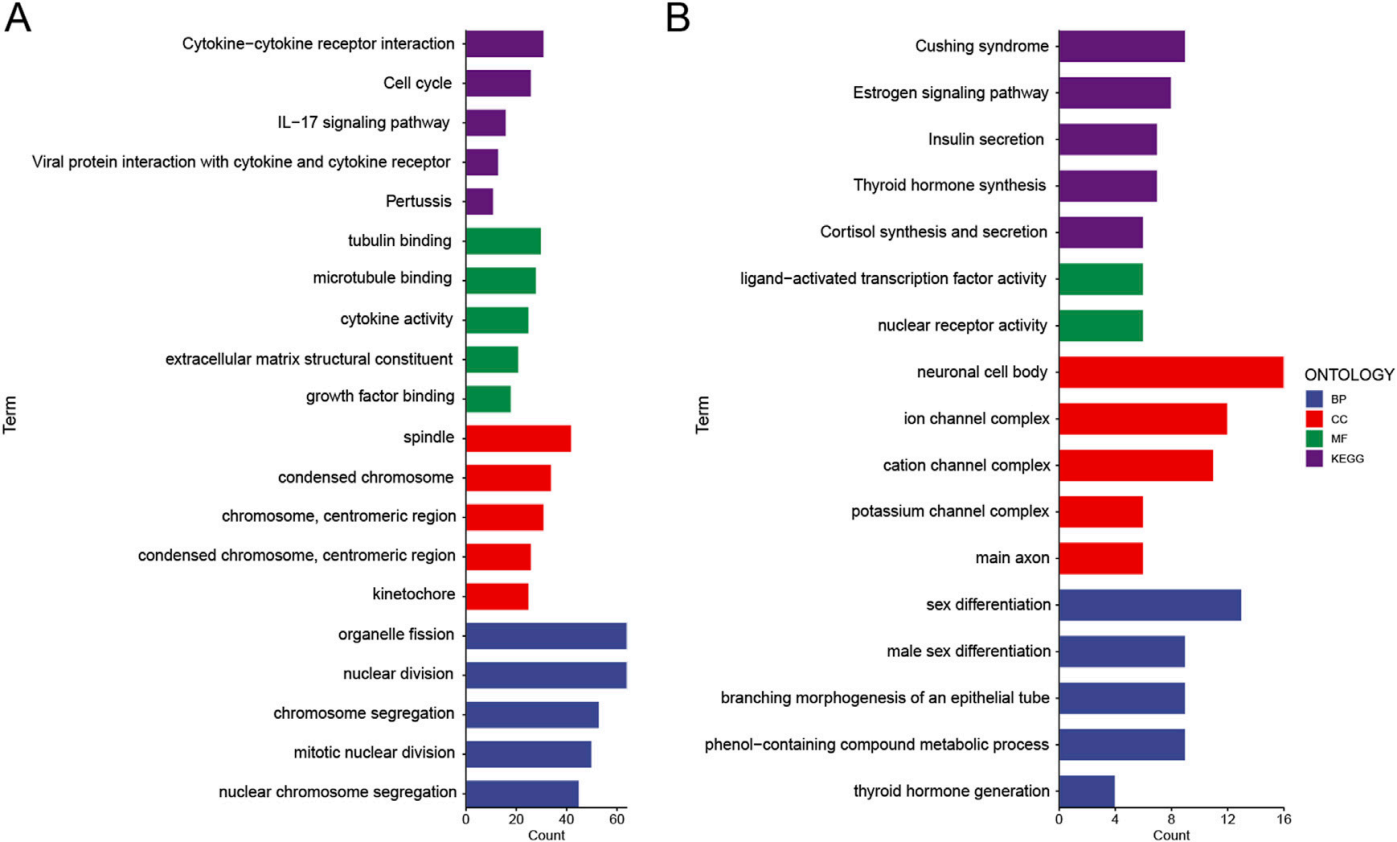
Functional analysis of upregulated and downregulated genes in the high epigenetic score group. **(A)** Functional analysis of upregulated genes in the high epigenetic score group was conducted. Biological processes (BP), cellular components (CC), molecular functions (MF), and pathways in the Kyoto Encyclopedia of Genes and Genomes (KEGG) were analyzed. **(B)** Functional analysis of downregulated genes in the high epigenetic score group was conducted. BP, CC, MF, and KEGG were analyzed.

In the high risk group, we found a series of functions enriched that are relevant to tumor invasion (Figure 6A). For molecular functions, we observed enrichment of tubulin binding, microtubule binding, cytokine activity, extracellular matrix structural constituent, and growth factor binding. For biological processes, we found enrichment of organelle fission, nuclear division, chromosome segregation, mitotic nuclear division, and nuclear chromosome segregation. Similarly, for cellular components, we saw enrichment of spindle, condensed chromosome, chromosome, and centromeric region. Furthermore, for KEGG pathways, we identified enrichment of cytokine-cytokine receptor interaction, cell cycle, IL-17 signaling pathway, viral protein interaction with cytokine and cytokine receptor, and pertussis.

In contrast, in the high risk group, we observed downregulation of functions related to metabolic regulation and differentiation, including KEGG pathways such as Cushing’s syndrome, estrogen signaling pathway, insulin secretion, thyroid hormone synthesis, and cortisol synthesis and secretion; molecular functions like ligand-activated transcription factor activity and nuclear receptor activity; cellular components such as neuronal cell body, ion channel complex, cation channel complex, and potassium channel complex; and biological processes including sex differentiation, male sex differentiation, branching morphogenesis of an epithelial tube, phenol-containing compound metabolic process, and thyroid hormone generation (Figure 6B).

In summary, tumors with higher epigenetic scores exhibited functional characteristics associated with cytoskeleton remodeling, cell cycle, cell division, and cell invasion, while those with lower epigenetic scores tended to be enriched for metabolic regulation and differentiation related functions. These findings provide new insights into the role of epigenetic regulation in tumor progression.

## Discussion

Epigenetic regulation plays a pivotal role in tumor initiation and progression due to its intricate mechanisms governing gene expression (Vogelstein et al., 2013). In recent years, a growing body of research has focused on uncovering the mechanisms of epigenetic regulation in cancer (Vogelstein et al., 2013). Building on this foundation, our study utilized bioinformatics approaches to systematically analyze the characteristics and potential significance of epigenetic regulation across pan-cancer. Using the TCGA dataset as a basis, we identified key epigenetic-related genes through comprehensive bioinformatics analysis. Leveraging these genes, we developed an epigenetic score and investigated its utility in assessing cancer patient prognosis by integrating clinical factors such as age and tumor type. Recent studies have made certain progress in exploring epigenetic regulation. For instance, Michael et al. (Cheng et al., 2023) used machine learning to investigate epigenetic factors in tumors, mapping the expression of epigenetic-related genes in highly invasive tumor cells using single-cell sequencing, but their study was limited to only 5 malignant tumors, including adrenocortical carcinoma (ACC), KIRC, lower grade glioma (LGG), liver hepatocellular carcinoma (LIHC), lung adenocarcinoma (LUAD). In contrast, we performed bulk RNA sequencing analysis across 33 malignant cancer types, offering a macroscopic view of epigenetic regulatory mechanisms. While this approach enables broad-scale insights into molecular alterations in epigenetic regulation, to some extent, it inherently lacks the specificity required for precise prognostic stratification of individual cancer types. Furthermore, Li Ding et al. combined single-nucleus RNA sequencing (snRNA-seq) with single-nucleus ATCA sequencing (snATCA-seq) to construct an integrative multi-omic atlas of 11 major cancer types. Their research indicated that some epigenetic drivers, like regulatory regions of *ABCC1* and *VEGFA*, appeared in pan-cancer, while some epigenetic regulators, like *FGF19*, *ASAP2* and *EN1*, and the *PBX3* motif, are cancer specific (Terekhanova et al., 2023). In our research, we only screened common epigenetic-related genes. We did not screen cancer specific regulators. As for signaling pathways and genes involved in epigenetic regulation, Li Ding et al. found that TP53, hypoxia, and TNF signaling were associated with cancer occurrence, while estrogen response, epithelial-mesenchymal transition, and apical junction were related to metastatic transformation (Terekhanova et al., 2023). This finding is consistent with our observation of abnormal estrogen signaling pathway expression in the high-risk epigenetic group.

Current research has focused on specific molecular mechanisms of epigenetic regulation. Yang et al. discovered that some m6A key regulatory factors (*ZC3H13*, *VIRMA*, and *PRRC2A*) have higher mutation rates in pan-cancer (Zhang et al., 2024). Hypermethylation of the *Per3* promoter was closely associated with tumor progression (Li et al., 2024). These findings provide important clues for understanding epigenetic regulation.

There are some limitations in our study. First, due to the difficulty in obtaining clinical tumor patient tissues, we could not evaluate the validity of the epigenetic score in external clinical datasets. Second, the incomplete treatment data for patients in the TCGA database limited our ability to further analyze the impact of epigenetic scores on drug treatment responses. As a bulk RNA sequencing analysis, we were unable to effectively distinguish the epigenetic characteristics of tumor cells from those of other cells in the tumor microenvironment, such as immune cells, stromal cells, and endothelial cells. In summary, our study offers a novel epigenetic score in cancer, laying the groundwork for future in-depth research.

## Conclusion

Collectively, our research provides novel insights into the intricate mechanisms of tumor development through the lens of epigenetic-related gene interactions. The innovative epigenetic scoring system developed in this study emerges as a potentially transformative tool for prognostic assessment across diverse cancer types. Moving forward, subsequent investigations should focus on rigorously validating these preliminary findings and exploring potential therapeutic strategies that target specific epigenetic regulatory pathways.

## Data Availability

The original data can be found at https://portal.gdc.cancer.gov/. The analyzed datasets are presented in the study/Supplementary Material. For further inquiries, please contact the corresponding authors directly.

## Supplementary material

Supplementary Figure S1 Epigenetic scores in pan-cancer at different stages or grades.

Supplementary Figure S2 The univariate Cox analysis of the epigenetic score in the training cohort and testing cohort.

Supplementary Figure S3 The relationship between epigenetic score, glycolysis, and EMT across pan-cancer.

Supplementary Figure S4 The relationship between epigenetic score, cell cycle, and DNA repair across pan-cancer.

Supplementary Figure S5 The relationship between epigenetic score, angiogenesis, and inflammation across pan-cancer.

Supplementary Table S1 Epigenetic gene list.

Supplementary Table S2 Fifty-nine candidate genes in epigenetic score.

## References

[B1] Angel C. Z. Stafford M. Y. C. McNally C. J. Nesbitt H. McKenna D. J. (2023). MiR-21 is induced by hypoxia and down-regulates RHOB in prostate cancer. Cancers (Basel) 15, 1291. 10.3390/cancers15041291 36831632 PMC9954526

[B2] Cai B. Song X. Q. Cai J. P. Zhang S. (2014). HOTAIR: a cancer-related long non-coding RNA. Neoplasma 61, 379–391. 10.4149/neo_2014_075 25027739

[B3] Cheng M. W. Mitra M. Coller H. A. (2023). Pan-cancer landscape of epigenetic factor expression predicts tumor outcome. Commun. Biol. 6, 1138. 10.1038/s42003-023-05459-w 37973839 PMC10654613

[B4] Dawson M. A. Kouzarides T. (2012). Cancer epigenetics: from mechanism to therapy. Cell 150, 12–27. 10.1016/j.cell.2012.06.013 22770212

[B5] Fang J. Zhang H. Jin S. (2014). Epigenetics and cervical cancer: from pathogenesis to therapy. Tumour Biol. 35, 5083–5093. 10.1007/s13277-014-1737-z 24554414

[B6] Han Y. Zhang Y. Y. Pan Y. Q. Zheng X. J. Liao K. Mo H. Y. (2023). IL-1β-associated NNT acetylation orchestrates iron-sulfur cluster maintenance and cancer immunotherapy resistance. Mol. Cell 83, 1887–1902.e8. 10.1016/j.molcel.2023.05.011 37244254

[B7] Hanahan D. (2022). Hallmarks of cancer: new dimensions. Cancer Discov. 12, 31–46. 10.1158/2159-8290.Cd-21-1059 35022204

[B8] Kong W. Yin G. Zheng S. Liu X. Zhu A. Yu P. (2022). Long noncoding RNA (lncRNA) HOTAIR: pathogenic roles and therapeutic opportunities in gastric cancer. Genes Dis. 9, 1269–1280. 10.1016/j.gendis.2021.07.006 35873034 PMC9293693

[B9] Li Y. Li W. Deng J. Yin M. (2024). PER3 promoter hypermethylation correlates to the progression of pan-cancer. Clin. Epigenetics 16, 140. 10.1186/s13148-024-01760-5 39402618 PMC11476066

[B10] Revathidevi S. Murugan A. K. Nakaoka H. Inoue I. Munirajan A. K. (2021). APOBEC: a molecular driver in cervical cancer pathogenesis. Cancer Lett. 496, 104–116. 10.1016/j.canlet.2020.10.004 33038491 PMC7539941

[B11] Romagnolo A. P. Romagnolo D. F. Selmin O. I. (2015). BRCA1 as target for breast cancer prevention and therapy. Anticancer Agents Med. Chem. 15, 4–14. 10.2174/1871520614666141020153543 25329591

[B12] Salgia R. Skarin A. T. (1998). Molecular abnormalities in lung cancer. J. Clin. Oncol. 16, 1207–1217. 10.1200/jco.1998.16.3.1207 9508209

[B13] Sanchez-Vega F. Mina M. Armenia J. Chatila W. K. Luna A. La K. C. (2018). Oncogenic signaling pathways in the cancer genome atlas. Cell 173, 321–337.e10. 10.1016/j.cell.2018.03.035 29625050 PMC6070353

[B14] Sapienza C. Issa J. P. (2016). Diet, nutrition, and cancer epigenetics. Annu. Rev. Nutr. 36, 665–681. 10.1146/annurev-nutr-121415-112634 27022771

[B15] Sharpless E. Chin L. (2003). The INK4a/ARF locus and melanoma. Oncogene 22, 3092–3098. 10.1038/sj.onc.1206461 12789286

[B16] Shilatifard A. (2012). The COMPASS family of histone H3K4 methylases: mechanisms of regulation in development and disease pathogenesis. Annu. Rev. Biochem. 81, 65–95. 10.1146/annurev-biochem-051710-134100 22663077 PMC4010150

[B17] Terekhanova N. V. Karpova A. Liang W. W. Strzalkowski A. Chen S. Li Y. (2023). Epigenetic regulation during cancer transitions across 11 tumour types. Nature 623, 432–441. 10.1038/s41586-023-06682-5 37914932 PMC10632147

[B18] Vogelstein B. Papadopoulos N. Velculescu V. E. Zhou S. Diaz L. A. Kinzler K. W. (2013). Cancer genome landscapes. Science 339, 1546–1558. 10.1126/science.1235122 23539594 PMC3749880

[B19] Zhang B. Hao Y. Liu H. Wu J. Lu L. Wang X. (2024). Interplay of RNA m(6)A modification-related geneset in pan-cancer. Biomedicines 12, 2211. 10.3390/biomedicines12102211 39457524 PMC11504890

[B20] Zhang L. Lu Q. Chang C. (2020). Epigenetics in health and disease. Adv. Exp. Med. Biol. 1253, 3–55. 10.1007/978-981-15-3449-2_1 32445090

[B21] Zhang M. Liu Z. Z. Aoshima K. Cai W. L. Sun H. Xu T. (2022). CECR2 drives breast cancer metastasis by promoting NF-κB signaling and macrophage-mediated immune suppression. Sci. Transl. Med. 14, eabf5473. 10.1126/scitranslmed.abf5473 35108062 PMC9003667

