# Supplementary figures and images for: Exploring the role of epigenetic regulation in cancer prognosis with epigenetic score
Source: Front Pharmacol. 2025 Feb 18;16:1538205. doi: 10.3389/fphar.2025.1538205 (PMC11876425; doi:10.3389/fphar.2025.1538205)

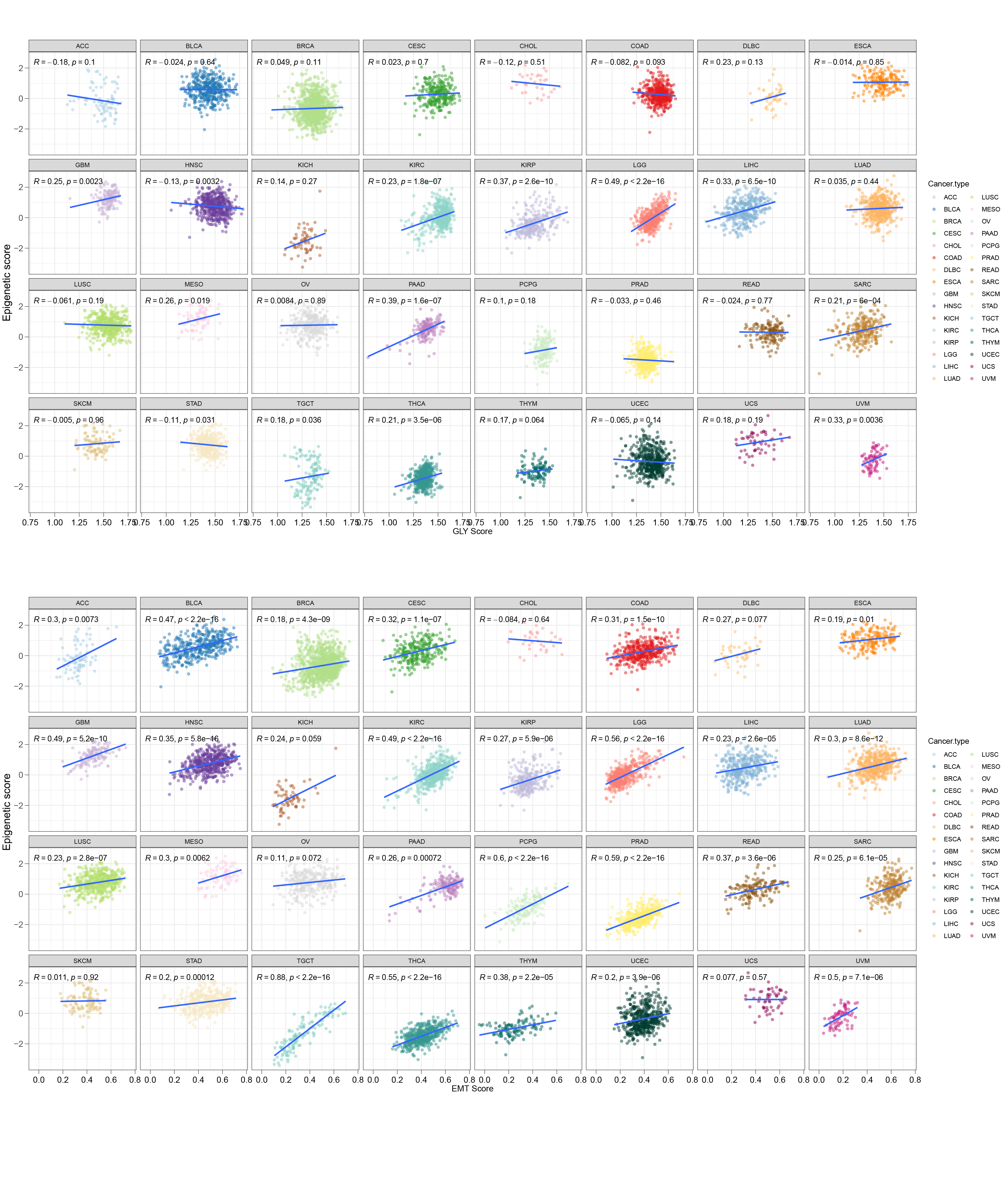

Supplement: Supplementary file 2 [file Image3.TIF]

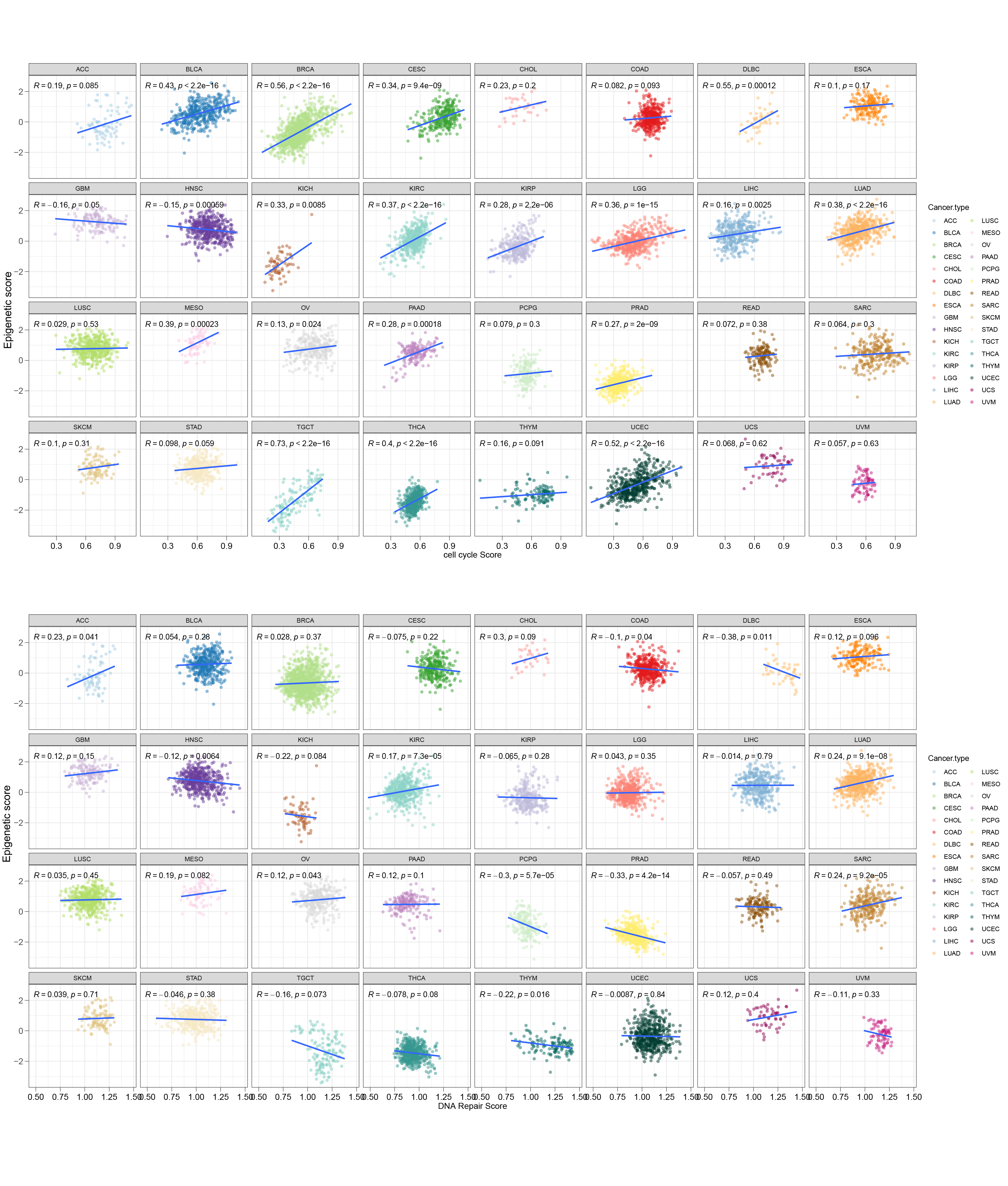

Supplement: Supplementary file 3 [file Image4.TIF]

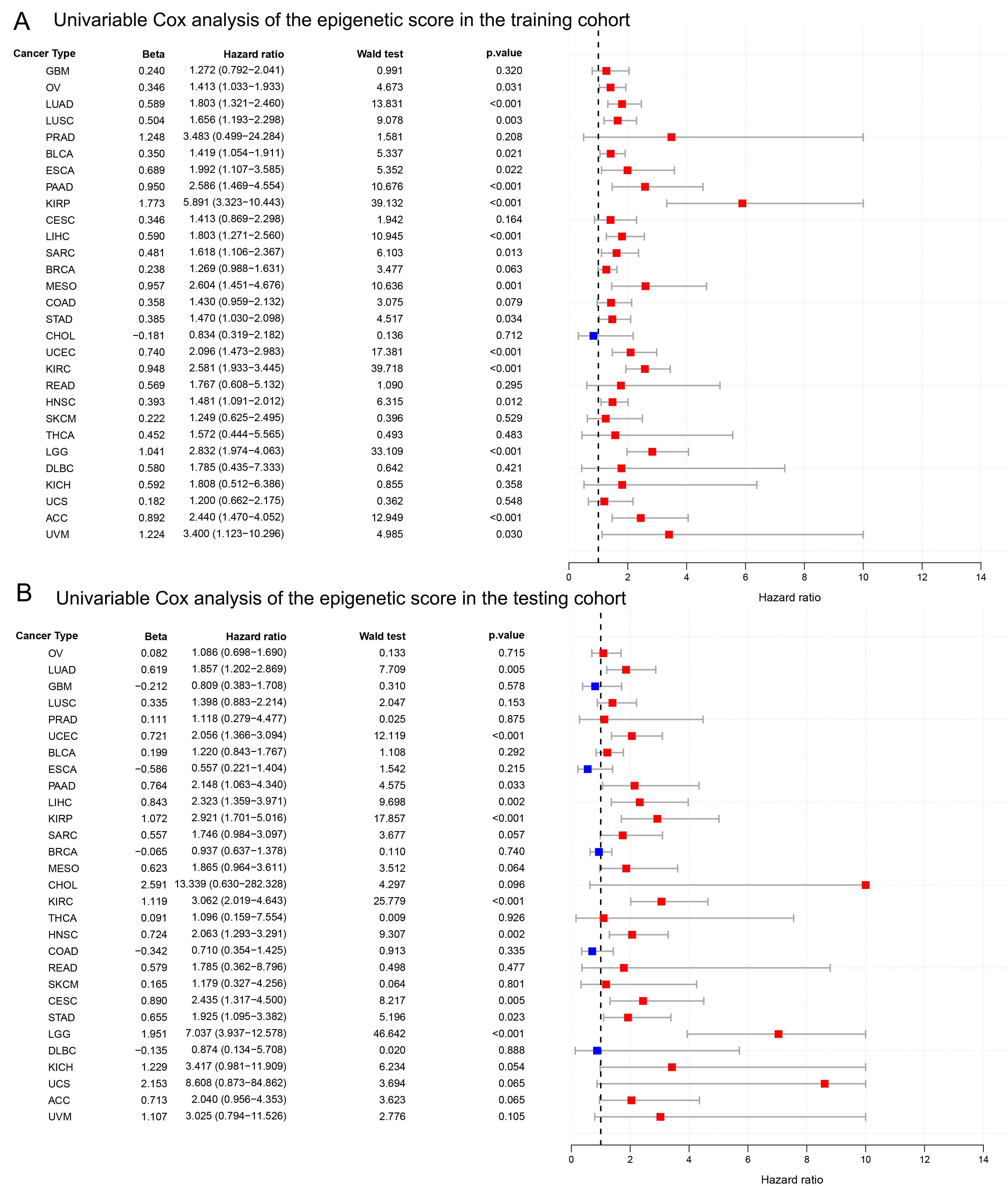

Supplement: Supplementary file 4 [file Image2.TIF]

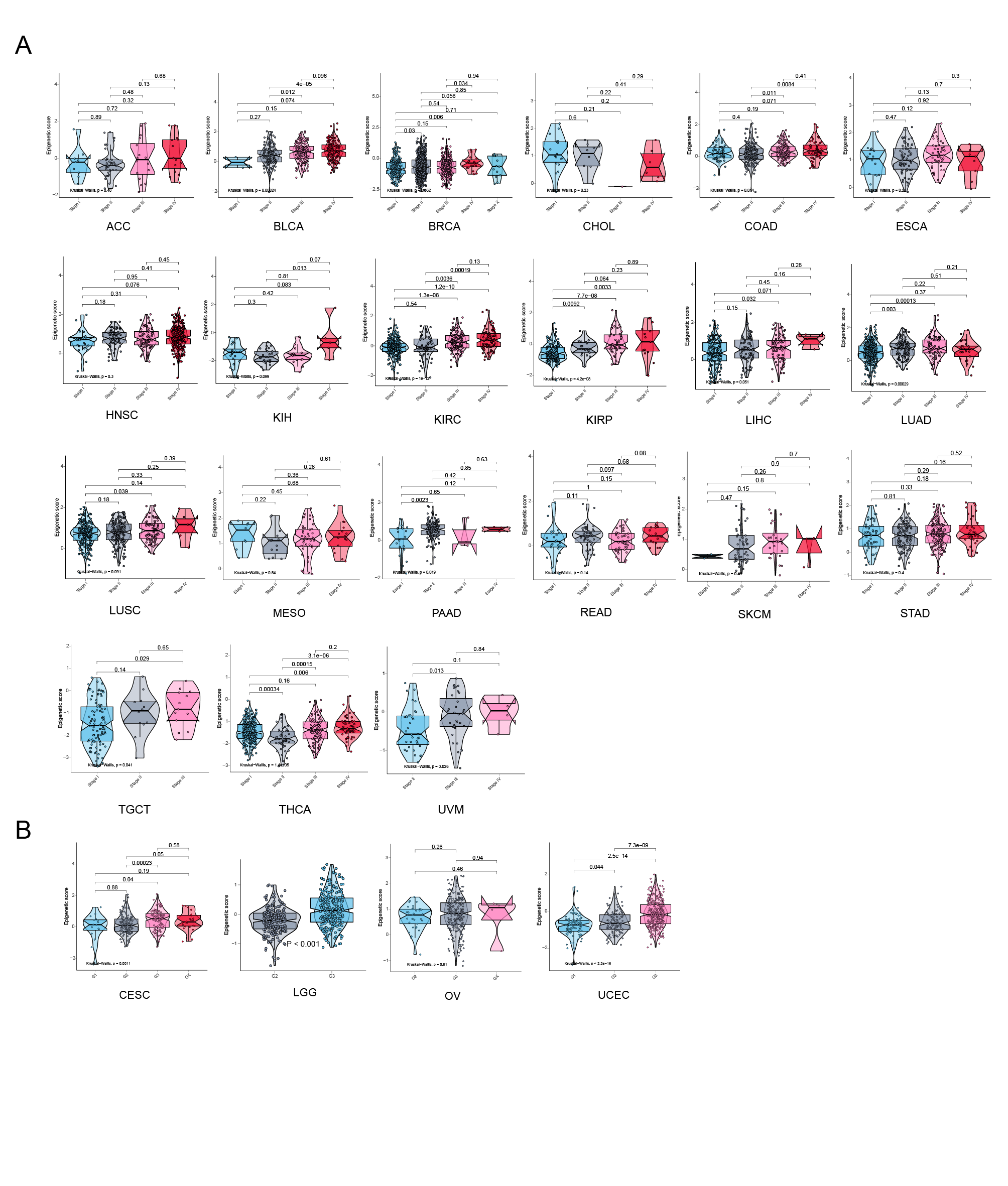

Supplement: Supplementary file 5 [file Image1.TIF]

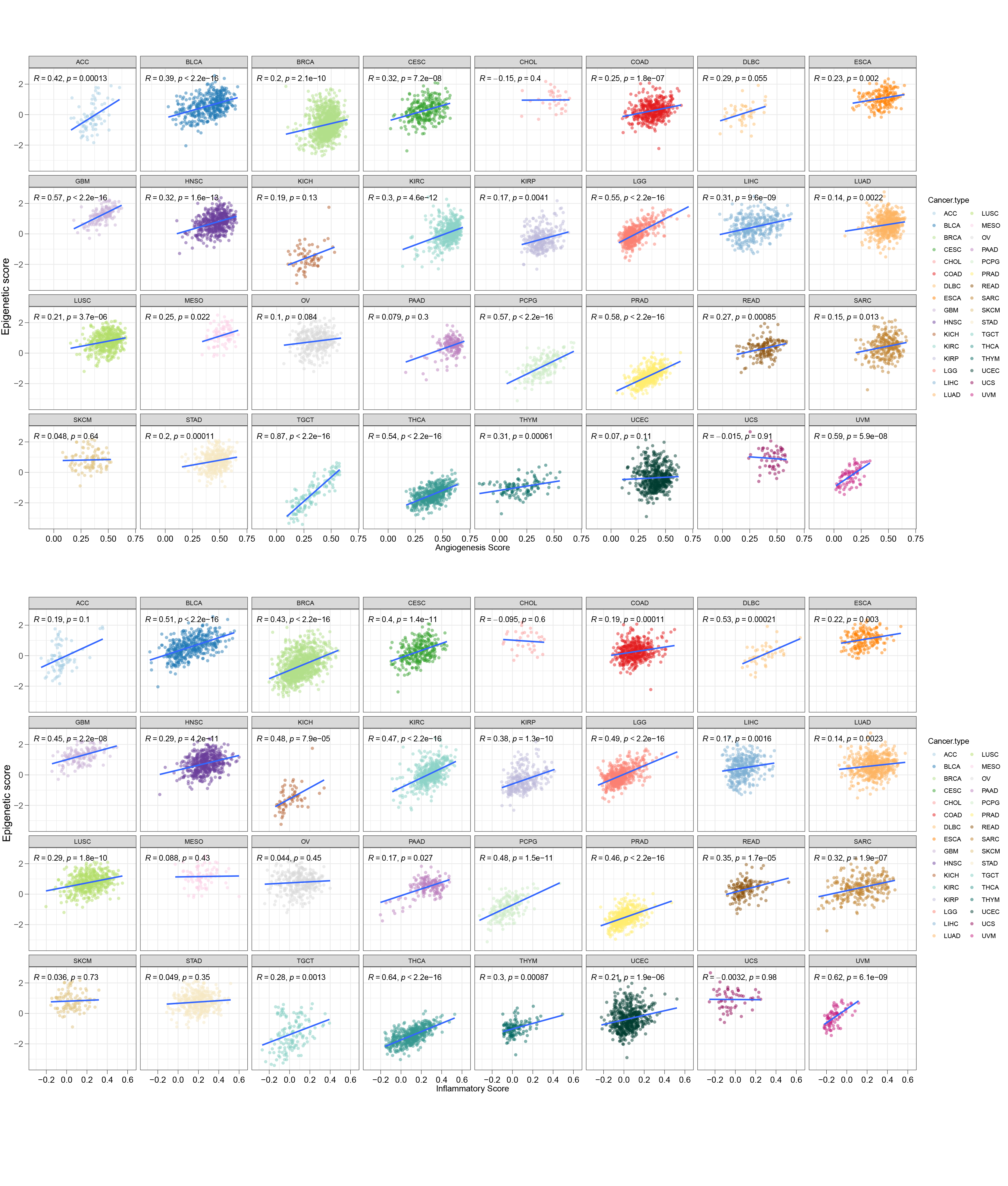

Supplement: Supplementary file 7 [file Image5.TIF]
